# From hijackers to architects: the role of prophages in bacterial genome evolution

**DOI:** 10.1128/jb.00458-25

**Published:** 2026-07-14

**Authors:** Lucía Rubí-Rangel, Josefina León-Félix, Claudia Villicaña

**Affiliations:** 1 Laboratorio de Biología Molecular y Genómica Funcional, Centro de Investigación en Alimentación y Desarrollo, Culiacán, Sinaloa, Mexico; 2 SECIHTI-Laboratorio de Biología Molecular y Genómica Funcional, Centro de Investigación en Alimentación y Desarrollo A. C., Culiacán, Sinaloa, Mexico; University of California San Francisco, San Francisco, California, USA

**Keywords:** prophages, genome plasticity, phage-host interactions, prophage induction, cryptic prophage, adaptation, domestication

## Abstract

Prophages, as the integrated or plasmid-like forms of temperate bacteriophage genomes, are critical genetic elements that establish stable interactions with their hosts, profoundly impacting host physiology and ecology. Prophages confer multiple advantages to their hosts. They promote changes in the bacterial genome structure and function—such as rearrangements, introduction of novel genes, and gene expression reprogramming—that often enhance bacterial fitness and persistence. Active prophages influence microbial community dynamics through the lytic-lysogenic cycle alternations, while both active and cryptic prophages modulate host behavior, genomic diversification, and interspecific interactions. Given that lysogeny is ubiquitous in nature, prophages play a pivotal role in shaping bacterial ecological functions and evolutionary trajectories. In this review, we provide a comprehensive overview of phage development and the contribution of prophages as modifiers of genome structure and function. We further discuss the long-term consequences of shaping genome architecture in the context of bacterial ecology and evolution, and propose emerging research directions to advance our understanding of phage-host interactions and their impact on microbial ecosystems.

## INTRODUCTION

Bacteriophages (phages) are one of the most important drivers of bacterial evolution, regulating physiology, genetic diversity, population dynamics, and coevolution through complex host-phage interactions within the microbial communities (1, 2). Phages exhibit four primary infection strategies: lytic, lysogenic, pseudolysogenic, and chronic infection cycles—the latter two being the least characterized (3, 4). Temperate phages alternate between lytic and lysogenic cycles. In the latter, lysogeny is established when the phage genome—referred to as a prophage—integrates into the bacterial chromosome or persists extrachromosomally as a phage-plasmid, whereas the host is then termed a lysogen (5).

Prophages typically follow one of two fates. First, active prophages may alternate between rounds of lytic and lysogenic cycles, influencing nutrient fluctuations and organic matter distribution across environments (6). Second, active prophages may acquire mutations in attachment sites or in genes encoding integrases, excisionases, or accessory factors required for excision, rendering them non-inducible. Throughout this review, the term “cryptic prophage” refers to these non-inducible forms, also known as grounded or defective prophages (7). Both active and cryptic prophages provide several advantages to their hosts, such as superinfection immunity against closely related phages, increased genetic variation, acquisition of novel traits, or the modulation of host gene expression (7). The underlying mechanisms may differ depending on the prophage type. Active prophages facilitate the acquisition of novel genetic material via transduction, modify genome structure through insertional mutagenesis upon integration, and promote recombination with various genetic elements within the bacterial genome. In contrast, cryptic prophages contribute to transduction only as genetic material mobilized by an active phage and modify the host genome structure only by recombination (8). In the long term, cryptic prophages may undergo genetic decay, primarily driven by deletions and substitutions that lead to genomic degradation and, ultimately, gene loss or domestication (9–11). During domestication, the host retains beneficial prophage-derived genes and mutations that enhance bacterial fitness and adaptation, shifting the interaction from a parasitic to a symbiotic relationship (9, 11).

Recent advances in genomic sequencing and comparative analysis have revealed that phages and phage-like elements are ubiquitous across bacterial taxa (12, 13), reinforcing their role in driving genetic and functional diversity. Although previous reviews have focused on lysogenic conversion, horizontal gene transfer, and phage-host dynamics, a comprehensive synthesis integrating the roles of active and cryptic prophages in genome remodeling, ecological adaptation, and long-term bacterial evolution remains limited. In this review, we provide an overview of lysogeny and the contribution of prophages as modifiers of genome architecture, functional diversification, and genetic innovation, discussing how these dynamic attributes shape phage-host interactions, with significant implications for bacterial ecology and evolution. To establish this integrative framework, we first examine the molecular basis of phage development, including lysogeny, prophage induction, and domestication, as key processes underlying their impact on bacterial genomes. In addition, we also integrate novel findings regarding the role of clustered regularly interspaced short palindromic repeats (CRISPR)-Cas and interactions with other mobile genetic elements (MGEs) that affect phage-host interplay by modulating their dynamics and driving coevolution.

## LYSOGENY AND PROPHAGE INDUCTION

The molecular basis of phage development focused on the establishment, maintenance, and induction of lysogeny is key for understanding the prophage impact on bacterial genomes. Phage infection begins with the host recognition and adsorption, mediated by specific interactions between the phage receptor-binding proteins and bacterial surface components, such as lipopolysaccharides (LPS), outer membrane proteins, peptidoglycan murein, flagella, fimbriae, capsular polysaccharides, and specific virulence factors (14). Following DNA injection, the phage commits to either the lytic or lysogenic pathway. In lysogeny, most temperate phages integrate into the bacterial chromosome, although others such as P1 and N15 can persist as autonomously replicating circular or linear plasmid-like elements (15, 16).

**Fig 1:**
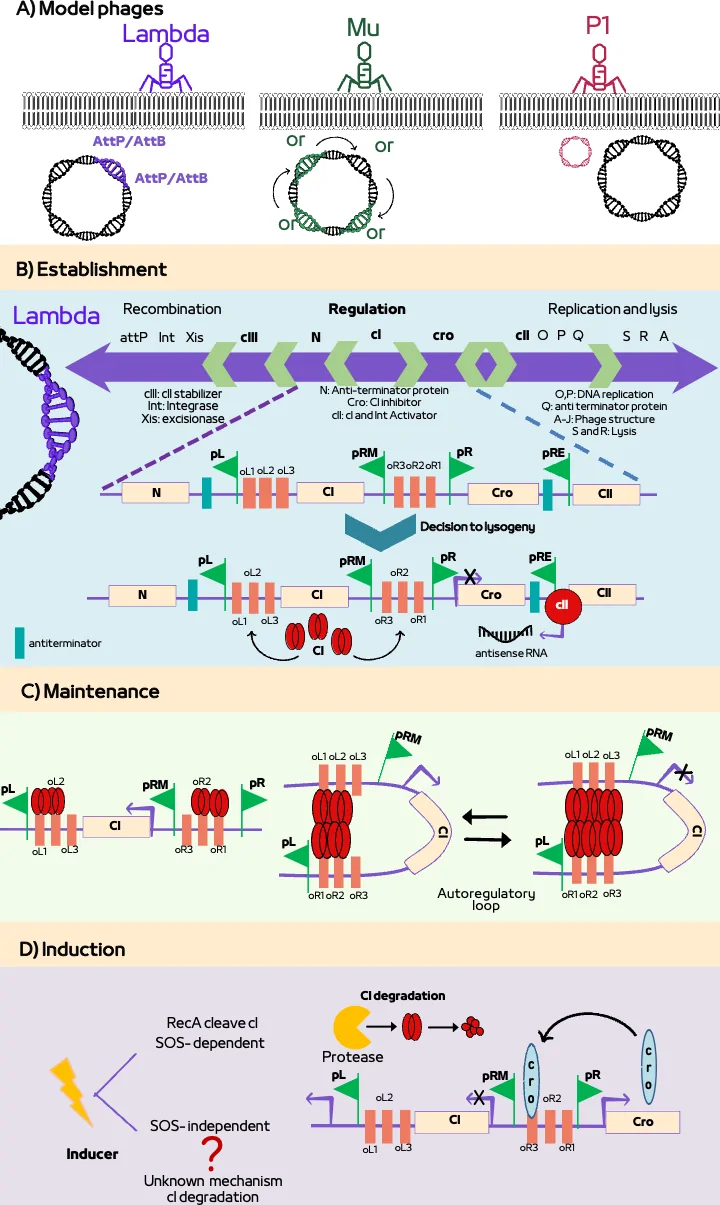
Typical phage models and common steps for the establishment of lysogeny. (**A**) Most lysogeny studies have been done in coliphages, including those that integrate in specific sites (λ), randomly (Mu), or remaining as episomes (P1) replicating autonomously as circular or linear plasmids. Using the λ phage as a model, the lysogenic cycle can be divided into three main stages: (**B**) Establishment. Transcription of the λ genome is modulated by the repressor CI and the anti-terminator protein N, using the host RNA polymerase. CI accumulates through the activation of the pRE promoter by CII, resulting in the transcription of *cI* and an antisense RNA that represses *cro*. Upon reaching threshold concentrations, CI activates its own transcription by binding to the pRM promoter and occupies the oL/oR operators to repress pL and pR promoters, triggering phage integration. (**C**) Maintenance. In the prophage state, *cI* expression depends completely on the pRM promoter. CI dimers bind to the oL1/oL2 and oR1/oR2 sites, interacting to form a large and stable DNA loop. The autoregulatory mechanism is driven by CI binding to oR2, which recruits RNA polymerase to pRM, stimulating its own expression. At high concentrations, CI binds to the low affinity oR3 and oL3 sites, leading to the repression of pRM. Conversely, when CI levels drop, oR3 and oL3 become free of the repressor and the CI synthesis resumes from pRM, until CI levels are restored to sustain lysogeny. (**D**) Prophage induction. Many extrinsic and intrinsic factors activate the lysogenic-lytic switch. Prophage induction involves the inactivation of the CI repressor via cleavage, leading to the de-repression of pR and pL promoters. Subsequently, pR directs the synthesis of Cro, which binds to oR3 to turn off *cI* expression from pRM, ensuring lysogenic to lytic transition. While CI inactivation is typically linked to the SOS response, other factors, such as N-acyl homoserine lactones (AHLs), can induce prophage excision through SOS-independent mechanisms that remain poorly understood. Figures were created with BioRender/Canvas.

The lysogenic cycle comprises three key stages: establishment, maintenance, and induction. In the classical phage λ model, the CI repressor plays a central role by binding the operator regions and silencing lytic promoters, thereby enabling prophage integration and maintenance (Fig. 1). Lysogeny is upheld through an autoregulatory feedback loop that ensures continuous CI expression. Induction occurs when environmental or physiological stresses inactivate CI and derepress lytic genes. The canonical mechanism for prophage induction is triggered upon SOS stress, which leads to CI cleavage in response to endogenous DNA damage, plasmid loss, or DNA-damaging agents (e.g., ultraviolet [UV] radiation) (17–19). Multiple environmental factors, including shifts in salinity, pH, temperature, the bacterial and phage densities, prophage-prophage regulation, the host metabolic state, quorum-sensing (QS) cues, and exposure to chemicals (e.g., mitomycin C or certain food- and drug-derived molecules) have been shown to promote induction (20–23). Additionally, SOS-independent pathways have been observed in response to QS signals (e.g., N-acyl homoserine lactones [AHLs], autoinducer-2 [AI-2], and quinolones), phenazines, elevated salt concentrations, or heat shock, although the underlying mechanisms remain poorly understood (20, 24–26).

Phage-derived signaling molecules were initially identified as modulators of lysis-lysogeny decisions. The arbitrium, a peptide-based phage communication system found in phages infecting *Bacillus*, allows phages to sense the density of lysogens and coordinate the lysogenic-lysis decision (27). This system relies on the propeptide AimP, which is secreted and processed by extracellular proteases to produce the mature arbitrium. At low AimP concentrations, AimR dimerizes and binds upstream of *aimX* to directly activate lytic development. After several rounds of viral replication, AimP is internalized by nearby cells and binds to AimR, causing the dimer dissociation. This inactivates *aimX* expression, promoting the shift toward lysogeny (28). Importantly, the *aimR* deletion triggered a low phage titer after mitomycin C treatment in *Bacillus* prophages, suggesting that the arbitrium system is essential for normal prophage induction (29). These findings demonstrate that lysis-lysogeny decisions are coordinated by phage-encoded communication systems that detect population-level parameters, integrating the physiological state of the host into this regulatory process.

Similar molecular mechanisms exist in other phages encoding CI-like repressors. In phage-plasmids P1 and N15, the linear dsDNA genomes circularize upon injection and maintain lysogeny via CI-like repressor, resulting in stable replicating extrachromosomal elements (30, 31). Like lambda, P1 and N15 phages are inducible by mitomycin C and UV radiation (5, 16). In gram-positive hosts, the lysogeny module often comprises CI-Cro homologous and other additional genes. In phage phi11 infecting *Staphylococcus aureus*, CI and Cro homologs regulate tripartite (O1, O2, and O3) and the P2 operator regions, respectively. In addition, the host LexA represses the phage L1 operator, modulating the expression of the antirepressor GP07. During lysogeny, CI binds to O1, O2, and P2 for the repression of *cro* and *gp07*, while LexA binding to P2 ensures the maintenance of the lysogenic state. Upon SOS response, CI and LexA undergo autoproteolysis, allowing the expression of Cro and GP07, where the latter binds to Cro to enhance its binding to O3 for CI repression, effectively switching to the lytic cycle (32).

CI-independent pathways have also been described involving the host LexA or the ImmR-ImmA system. In extrachromosomal tectiviruses, LexA interacts with phage-encoded Gp7, and the resulting complex directly represses promoters (P1, P2, and P3) required for lytic development. Upon DNA damage, LexA is autoprocessed, and once its concentration drops below a threshold, P1 and P2 are derepressed, triggering the lytic cycle (33). The ImmR-ImmA system, found in phages and integrative and conjugative genetic elements, comprises the transcriptional repressor ImmR and the site-specific protease ImmA. In phage Φ105, ImmR maintains lysogeny by repressing the PM and PR promoters. Upon a RecA-dependent SOS response, ImmA is activated and cleaves ImmR, initiating the switch to the lytic cycle (34). Beyond the consequences mediated by lysis-lysogeny alternancy, prophages exert broader effects on their hosts and extend across strains and populations via horizontal gene transfer (HGT)—for instance, through the mobilization of MGEs (35). Thereafter, several mechanisms contribute to the remodeling of the structure and function of bacterial genomes, as discussed in detail in the following sections.

## IMPACT ON THE BACTERIAL GENOME

### Genomic plasticity and structure

Bacterial genomes are highly plastic due to their ability to undergo structural and functional changes in response to environmental selective pressures. Genomic plasticity is influenced by several factors such as HGT, recombination, chromosomal rearrangements, and mutation, processes often mediated by prophages. In prokaryotes, genetic acquisition involves mechanisms such as gene duplication and HGT, the latter primarily facilitated by phage transduction (36). The different modes of transduction (generalized, specialized, and lateral) are responsible for the gene exchange among strains and species, mobilizing chromosomal regions, phage-inducible chromosomal islands (PICIs), and small plasmids (e.g., pI258 and pC221) (13, 35, 37) (Table 1). In fact, lateral transduction has shown higher transfer frequency rates than generalized transduction, conjugative transposons, and plasmids in *S. aureus* and *Staphylococcus enterica* (38), suggesting it as an efficient process for the exchange of immobile chromosomal genes. Thereby, transduction largely contributes to the intra- and interspecific dissemination of advantageous genes and MGEs, promoting host survival and phage persistence under rapidly changing environments.

**TABLE 1:** Examples of the impact of prophage-host association with the genome size and structure

Biological impact	Mechanism	Effect	Example	Reference
Genome size	HGT mediated by phage and PICI transduction	Expansion	Phage 80α and SaPIs (SaPI1 and SaPIbov2) can disseminate plasmids (pI258 and pC221) via general transduction in *S. aureus*.	(35)
			Using CPRINS-FISH, phages P1, P4, and EC10 were able to transfer a GFP-containing plasmid indicator to *Enterobacteriaceae*, being transductants only those plaque-forming strains. Moreover, EC10 was able to transduce the plasmid into natural bacterial recipients from freshwater environments.	(37)

	Domestication of prophages	Expansion	Genomic analysis of prokaryote genomes indicates that bacterial genomes were larger when associated with viruses and plasmids.	(39)
			In Enterobacteria, prophage domestication involves a rapid inactivation and slower genetic degradation, giving a gain of genetic material. Accessory regions and proteins encoded for phage function can be selected in the long term.	(9)
	Antiphage and defense systems	Less acquisition of genome diversity	Genomic analysis showed that genomes with CRISPR systems were significantly more reduced, showing a preference to target viruses over plasmids.	(39)
			PICIs encode several defense system proteins, which are disseminated by helper phages, used to compete against phages and other MGEs to protect the helper phage and the bacterial host.	(40)
			A prophage encodes the phage defense BstA protein that suppresses lytic replication of foreign phages by abortive suppression in Gram-negative bacteria.	(41)
			Some defense systems, including CRISPR-Cas, were associated with reduced gene gain rates, smaller pangenomes, and fewer phage-related genes.	(42)
			P4-like satellites and P2 phages were identified as hotspots to harbor a diversity of arrays of antiphage proteins, some encoding type II and III-restriction modification systems and retrons, and proteins with unknown functions. Validation of several unknown proteins demonstrated an increase in resistance to phages.	(43)
Genome structure	Recombination	Insertion	*E. coli* recombination was demonstrated experimentally for attC sites derived from environmental virulent phages and bacterial class one integron-integrase (IntI1), revealing the potential of phages to integrate into integrons and vice versa.	(44)
			Temperate lamboid phages recombined with cryptic prophages by homologous recombination, promoting mosaicism. This process was mediated by Rad52-like recombinases encoded by phage, promoting gene shuffling among temperate phages and other MGEs.	(8)
		Rearrangement and hybrid viruses	Sporulation induces SPB prophage recombination with indigenous phi3T in *B. subtilis,* resulting in a new hybrid prophage in a different location which can produce and release virulent phage hybrids.	(45)

			Generation of hybrid CTX virus by recombination with indigenous prophage and RS1 element in *V. cholerae*	(46)
		Rearrangement and inversions	Syntenic block analysis revealed genomic rearrangements (including inversions) in *Neisseria gonorrhoeae* and *N. meningitidis* genomes, revealing that some rearrangements were associated with several MGEs, including several prophages (Nf1–Nf3 and NgoΦ1–NgoΦ9) and Mu-like prophages Pnm1, Pnm2, and Mu MenB.	(47)
		Deletion	Imprecise excision of ΦAP1.1 prophage leads to deletion of spacers in the CRISPR locus in *S. pyogenes*. Strikingly, deleted spacers were packaged into viral particles and transferred to new hosts, spreading immunity.	(48)
	Imprecise prophage excision	Loss of function of phage repressor	Insertions and deletions within a ~1,000 bp region encoding a c1-type phage repressor were detected in a hybrid phage-plasmid infecting *Tritonibacter mobilis*. These mutations could be induced by replication slippage. Mutations of c1 repressor occurred Indels when the bacterial population reached 40 generations to switch toward productive infection. Loss of phage repressor together segregationally drifts control phage-plasmid dynamic transmission through generations.	(49)


Once established, prophages may trigger intragenomic changes through mutation and recombination. Mutation rates often increase during host-phage coevolution; meanwhile, prophages act as mutational hotspots during lysogeny (50). They accumulate insertions/deletions (indels) and point mutations that cause small-scale changes, which can subsequently facilitate large-scale rearrangements (47). Furthermore, chromosomal rearrangements also occur upon prophage integration and through recombination with superinfecting phages, resident prophages, and plasmids (8, 45, 51). These rearrangements are commonly flanked by prophage sequences (47, 52, 53), suggesting that they serve as recombination hotspots potentially enhanced by phage-encoded recombinases (8). Such events give rise to genome plasticity regions, which comprise large hypervariable segments typically enriched with MGEs and adaptive genes (e.g., stress resistance, virulence, and metabolic genes), which enable rapid responses to environmental challenges (54). In phage-plasmids, recombination events with plasmids or phages increase the transfer of core genes, defense systems, and antibiotic resistance genes via both horizontal and vertical transmission (51). Moreover, recombination in P1-like plasmids facilitates their interconversion between integrative prophages and independent plasmids, a process often triggered by gene loss, which significantly impacts the spread and evolution of these MGEs (51).

Structural changes are promoted by homologous, site-specific, or illegitimate (non-homologous) recombination. Typically, homologous recombination occurs between prophages sharing genomic regions with at least 77% identity, driving mosaicism in cryptic prophages (8). This process enhances diversification through module swapping (7), gene shuffling (e.g., *psbA* in cyanobacteria) (55), the creation of hybrid prophages (45), and hypothetically, the reactivation of cryptic prophages via restoration of the excision module. Additionally, site-specific recombination is well-characterized in lambdoid phages (e.g., lambda or P22) involving the *attP* and *attB* sites (56). Recent evidence indicates that virulent and temperate phages carrying integrons can also recombine with bacterial integrons via *attC* sites, representing a novel route for lateral dissemination across bacterial lineages (44). Finally, illegitimate recombination between unrelated sequences, such as random prophage transposition, may generate novel prophages and contribute to specialized transduction (45). Generally, recombination can induce mutagenesis by disrupting genes or regulatory elements and prompt structural changes (e.g., gene duplications) (7). As mutational hotspots, cryptic prophages are prone to accumulating mutations over time (9), which is hypothesized to serve as buffer regions that reduce the risk of lethal mutations in essential host genes (7). Taken together, prophages function as genomic catalysts that continuously reshape bacterial chromosome architecture, generating the structural variation upon which natural selection acts.

Genomic plasticity facilitates rapid pathogen adaptation by increasing genetic and phenotypic diversity during infections (57). Similarly, recombination between prophages leads to large-scale genomic rearrangements that enhance diversity and fitness (58). Notably, these genetic exchanges and mutations can also be adaptive for the phage by promoting tail fiber alterations, evasion of the host immune system, expansion of the host range, or the modulation of productive infections in response to bacterial proliferation (48, 49, 58, 59). Although prophage maintenance is costly to the host, genomic studies suggest that phages drive genome expansion, as evidenced by the correlation between a higher prevalence of plasmids and viruses and larger bacterial genomes (39). In this context, genome expansion enlarges the genetic pool upon which selective pressures act to preserve and optimize genes, thereby enhancing novel functions and regulatory diversity while balancing the metabolic burden of prophage maintenance with the adaptive benefits to the host (7). Thus, prophage-driven genomic plasticity should be understood as an adaptive force that simultaneously broadens bacterial genetic diversity and accelerates genome evolution across ecological timescales. The described structural remodeling represents only a part of the prophage impact on bacterial biology. Prophages also expand the functional and regulatory repertoires of their hosts through the acquisition of novel genes and the modulation of gene expression.

### Functional and regulatory impacts of prophages

#### Acquisition of novel genes and functional innovation

Prophages expand the functional capabilities of their hosts through the acquisition and generation of novel genes. These genes are diverse, including morons, auxiliary metabolic genes (AMGs), and others lacking defined classification (Table 2). During lysogeny, these genes can confer novel phenotypes leading to lysogenic conversion (52), a process commonly associated with the shift of non-pathogenic bacteria into pathogens, although this also occurs in other symbiotic interactions (60).

**TABLE 2:** Examples of the impact of prophage-host interactions in the context of gene function

Biological impact	Mechanism mediated by	Effect	Examples	Reference
Functional diversity	Morons	Novel functions	Toxins encoded by prophages harbored by several pathogens such as *E. coli*, *Corynebacterium diphtheriae, Clostridium botulinum, Staphylococcus*, and *Vibrio cholerae*	(52)
			The DLP4 phage infecting *Stenotrophomonas maltophilia* carried the *folA* gene encoding dihydrofolate reductase conferring resistance to trimethoprim, and *ybiA* encoding N-glycosidase involved in swarming motility.	(61)
			Bioinformatics survey revealed that phage-plasmids are rich in antibiotic resistance genes (ARGs). Five prophages carrying ARGs were experimentally induced with mitomycin C and capable of infecting and converting other *E. coli* strains.	(5)
			RSY1 phage that infects *Ralstonia solanacearum* has a type VI secretion system	(62)

			A cryptic prophage encodes the superoxide dismutase SodCI in *S. Typhimurium*.	(63)
			A prophage-related protein regulates growth and swimming motility in *V. parahemolyticus*.	(64)
			In the gut commensal *Bacteroides* spp., the Bxa toxins are encoded by phages and exhibited antibacterial ADP-ribosyltransferase activity. Moreover, Bxa targeted non-muscle myosin II in epithelial cells triggering the remodeling of the actin cytoskeleton and inosine secretion, which aids in gut colonization.	(65, 66)
			In marine sponges, an ankyrin-domain-containing protein increased bacterial survival by reducing phagocytosis by the eukaryotic host.	(67)
	AMGs	Modulation of host metabolism	Fecal microbiome from infants and adults was rich in genes involved in carbohydrate metabolism and degradation, amino acid metabolism, and carbon metabolism.	(68)
			Lytic viruses exhibit a greater AMG diversity, encoding proteins for boosting their replication; meanwhile, temperate phages encode AMGs for host survival.	(69)
			In benthic and pelagic samples, genes encoding methyltransferases, photosynthesis proteins, 7-cyano-7-deazaguanine synthesis, and cobalamin biosynthesis were abundant.	(70)
			Lysogenic phages were enriched in highly contaminated environments with chromium and contained AMGs related to heavy metal detoxification.	(71)
			Novel AMGs participating in folate and nucleotide metabolism in bathypelagic microbiomes, where water age was determinant for virome composition showing a higher AMG content in older water masses.	(72)
	Other phage-encoded genes	Dual function	Prophage phiRv2 gene *Rv2650c* predicted as a capsid enhanced resistance to *M. smegmatis* to macrophages and oxidative stress, surfactant, and acid environment. Moreover, Rv2650c protein suppresses the secretion of pro-inflammatory cytokines	(73)
			An active prophage in *Streptococcus mitis* encodes PblA and PblB proteins, which promote binding to human platelets acting as adhesion molecules.	(74)
		Repurposed function	A phage lysin gene, initially toxic, was integrated into the bacterial genome and became part of the SpmX protein. This modified lysin domain is essential for cell shape and developmental regulation in Caulobacterales.	(11)
	Host and MGE encoded genes	Virulence spreading	In clinical *S. aureus* strains, SaPIs were disseminated by endogenous prophages.	(75)

		Acquisition of novel defense systems	Imprecise excision of ΦAP1.1 prophage leads to deletion of spacers in CRISPR locus in *S. pyogenes*. These spacers were packaged into viral particles and transduced to new hosts.	(48)

Morons are non-essential phage genes that provide accessory functions to enhance bacterial fitness. These genes encode proteins involved in key pathogenic processes, enable competitive features (e.g., killing competitors or competing for public goods), increase survival, or mediate mutualistic interactions (e.g., ankyrin proteins and Bxa toxin) (52, 53, 65–67). Moreover, certain morons confer resistance to phage infection through superinfection exclusion proteins, providing a competitive advantage to lysogens over non-lysogens (76). In addition, AMGs were anciently acquired from bacterial hosts via HGT, allowing phages to reprogram host metabolism to either enhance viral replication or boost host fitness under environmental stress. These genes encode proteins involved in pathogenicity, nutrient acquisition, photosynthesis, motility, biofilm formation, and transport, thereby shaping community metabolism and structure (69, 70, 77). For instance, AMGs are prevalent in marine and contaminated habitats, where they modulate biogeochemical cycles, primary production, and host survival (71, 72, 78). Interestingly, metagenomic studies reveal that AMG diversity is higher in lytic than temperate phages, suggesting that AMG composition is highly lifestyle-dependent. Lytic viruses mainly contained AMGs enhancing viral replication (e.g., cofactor and vitamin metabolism, lipid metabolism, signaling proteins, antioxidants, and chaperones); meanwhile, temperate phages harbored AMGs that augment host fitness (e.g., defense systems, pathogenicity, stress responses, and cell growth) (69). Finally, other prophage genes, such as structural genes encoding capsids and tails, contribute to host fitness by improving adhesion and resistance to adverse conditions (73, 74). Prophage-encoded genes function as active contributors to host metabolism, ecological competitiveness, and environmental adaptation, with their functional impact shaped by the lifestyle of the phage and the selective pressures of the habitat.

Prophages also drive gene diversification and innovation through domestication and recombination. Compelling evidence demonstrated that the SpmX protein from Caulobacterales is the result of the domestication of a phage-derived toxic lysin co-opted to regulate cell morphology and development (11). Genomic analysis of the photosynthetic *pbsA* gene suggests that phage-mediated recombination between *Synechococcus* and *Prochlorococcus* facilitated the acquisition, reshuffling, and exchange of *pbsA* genes, promoting their diversity and evolution (55). Furthermore, prophages promote mosaicism and the emergence of hybrid genes through recombination with other prophages or the bacterial chromosome (45, 46).

These mechanisms of gene exchange, diversification, and innovation allow lysogens to outcompete non-lysogens, kill competitors, avoid secondary viral infections, or mediate tripartite interactions of bacterial hosts with eukaryotic symbionts and associated microbiota (65, 67, 71, 72, 79). Such interactions influence host fitness and prophage persistence, facilitating niche specialization (71, 72). Overall, prophage-mediated gene acquisition and diversification represent major mechanisms of functional innovation in bacteria, enabling rapid phenotypic expansion without the exclusive dependence on *de novo* mutations. Beyond these contributions to functional diversification, prophages also influence host biology through the modulation of gene expression, although this impact remains underestimated because most prophage genes lack functional annotation.

#### Gene regulation

Prophages actively participate in the regulation of host gene expression and metabolism through multiple mechanisms (Table 3). Structural rearrangements and prophage integration alter the integrity and activity of genes, promoters, and other regulatory regions; simultaneously, the transcription of phage-encoded regulators (e.g., repressors, activators, transcription factors, or small non-coding RNAs [sRNAs]) modulates host gene expression. A consequence of prophage integration is the disruption of genes or *cis-*regulatory regions, causing loss of function or metabolic reprogramming (e.g., phage promoters in frame with host genes) (80, 81). Prophage integration can also disrupt bacterial immunity. In *Streptococcus pyogenes*, integration of ΦAP1.1 into the CRISPR locus causes the selective impairment of spacer transcription and crRNA processing (48).

**TABLE 3:** Examples of the impact of prophage-host interactions in the context of gene regulation

Biological impact	Mechanism mediated by	Effect	Examples	Reference
Gene regulation	Gene disruption	Loss of gene function by integration	Integration of ECA29 prophage disrupted the *pflA* gene, which is involved in anaerobic metabolism; a reduction in motility and virulence in potato was observed upon prophage deletion, suggesting that ECA29 may regulate virulence in *Pectobacterium atrosepticum*.	(82)
	Phage promoter transcription	Metabolic reprogramming	Temperate phage HK022 integrated into *E. coli* genome reprogrammed an anaerobic respiratory system through upregulation of the host-encoded *torS* signal protein by replacing the native promoter with a phage promoter.	(81)
	Active lysogeny	Reversible active lysogeny	Excision of Φ10403S prophage was excised upon phagocytosis of *L. monocytogenes*, restoring a functional *comK* required for phagosomal escape. After excision, prophage remained as an episome and did not produce virions nor bacterial lysis. Eventually, phage genome is inserted disrupting *comK* again.	(79)
			In *S. pyogenes*, the prophage remnant SpyCIM1 remains as a replicative episome during bacterial exponential growth. Under stationary growth phase, SpyCIM1 is integrated between *mutS* and *mutL,* disrupting the MMR system to increase mutation rate and enhance probability of survival. Once bacteria return to exponential growth, SpyCIM1 is excised again, leaving a functional MMR system.	(83)
		Non-reversible active lysogeny	SPβ and *skin* excision in *B. subtilis* allowed reconstitution of *spsM* and *sigK* genes, respectively, which are essential to initiate and complete the sporulation process. Excised elements did not reintegrate and are eventually lost in the dying mother cell.	(84, 85)
	Prophage-encoded repressors/inhibitors	Inhibition of QS	Phage DMS3 encodes a QS anti-activator protein, Aqs1, that inhibits the master regulator LasR and PilB. PilB blocks superinfection by phages.	(86)
		CI repressor	The CI repressor encoded by lambda phage binds directly at the promoter of *pckA* (phosphoenolpyruvate carboxylase) gene for repression triggering a slow growth.	(87)
	Transcription factors and regulators	YdaT	YdaT regulates positively the host *rcsA* gene, increasing RcsA impacts the RcsA/B regulon affecting motility, capsule biosynthesis, biofilm formation, and cell division.	(88)
		AppY	Transcriptional regulator that modulates the expression of more than 200 genes in *E. coli* K-12 affecting motility, biofilm formation, and resistance to harsh conditions	(89)
		AlpA	AlpA is encoded by the CP4-57 cryptic prophage in *E. coli*. AlpA repressed PhoR, a positive regulator of phoB and pstB, triggering a reduced persister cell resuscitation. Deletion of cryptic prophage increased persister resuscitation by increasing phosphate signaling mediated by activation of PhoR. This shows that prophage controls persister cell resuscitation via phosphate uptake to prevent it in the absence of nutrients	(90)
		DicC	DicC, carried by the cryptic Qin prophage in *E. coli*, regulates hundreds of genes across the host genome, affecting genes associated with acid and oxidative stress. Moreover, specific *dicC* induction gives rise to growth defects in the host.	(91)
		Cro	During lysogeny, Cro activated the expression of the EHEC type III secretion system, enhancing the virulence of different outbreak *E. coli* strains.	(92)
		Sigma factors	Phages capable of infecting spore-forming hosts encoded sporulation-like sigma factors homologous to *sigF* and *sigG*. Expression of prophage-encoded sigma factors in *B. subtilis* reduced spore production.	(93)
		SrpA	Protein encoded by phage K5 infecting *P. aeruginosa*, which is predicted to bind to 66 sites proximal to promoters. By transcriptomic approaches, SrpA directly or indirectly regulates diverse genes involved in basic cellular processes such as motility, biofilms, pyocyanin synthesis, and chemotaxis.	(94)
	Prophage-encoded RNAs	sRNA DicF	The sRNA DicF, encoded by the cryptic lambdoid prophage Qin in several *E. coli* genomes, was shown to regulate *ftsZ* required for cell division. Moreover, DicF repressed translation of *xylR* and *pykA*, involved in xylose and pyruvate metabolism, respectively.	(95)

Leveraging the dynamic nature of active prophages, many bacteria have evolved a regulatory switch based on precise prophage excision and subsequent reactivation of host genes, a process termed active lysogeny (96). “Reversible” active lysogeny is well-characterized in *L. monocytogenes* and *S. pyogenes*, in which the excised prophages remain as episomes, allowing timely and synchronized gene modulation depending on environmental cues or pathogenic development; eventually, prophage reintegration occurs, restoring the original genomic configuration (79, 83). In contrast, in “irreversible” active lysogeny, prophage reintegration does not occur. For instance, the *skin* remnant prophage is excised to regulate gene expression at late stages of sporulation in *B. subtilis* and is subsequently lost in the mother cell (84, 85). Due to the lack of phage production or bacterial lysis, active lysogeny is suggested to be an adaptive behavior suited to the lifestyle of the host. In broader terms, active lysogeny exemplifies how prophages have been co-opted as precision regulatory switches, enabling bacteria to reversibly modulate gene expression in a stimulus-dependent manner without incurring the costs of phage production.

Prophages also encode a diversity of regulatory elements acting in *trans*. Many regulatory proteins coordinate the expression of morons, AMGs, and genetic components of the lysogenic cycle. However, they can also directly modulate bacterial gene expression, including virulence factors, metabolic pathways, and QS regulators (81, 86). Indeed, CI and Cro modulate genes involved in metabolic reprogramming and virulence, respectively (87, 92). Others, such as sigma factors and transcriptional regulators (e.g., SrpA, AppY, DicC, and YdaT), have been shown to regulate hundreds of bacterial genes affecting motility, biofilm formation, growth, virulence, and resistance to harsh conditions (88, 89, 91, 93, 94). Furthermore, phage-encoded sRNAs have functions analogous to eukaryotic microRNAs, regulating gene expression by degrading messenger RNAs or repressing translation. These sRNAs affect viral processes, such as DNA replication and the decision between lysogenic and lytic cycles, as well as bacterial functions including colonization, metabolism, motility, and virulence (27, 95, 97). This regulatory diversity, in conjunction with the novel gene repertoires, renders prophages active components of the bacterial genome. Prophages profoundly affect host physiology and interactions, consequently altering bacterial fitness and serving as critical drivers shaping the host ecology and evolution. The regulatory and functional contributions determine the retention, inactivation, or elimination of prophages throughout time, impacting host physiology and their long-term evolutionary pathway. This demonstrates that prophage persistence is far from neutral but is instead influenced by a balance of related costs and context-dependent benefits.

## PROPHAGE MAINTENANCE, LOSS, AND DOMESTICATION

Prophage maintenance may impose a significant metabolic burden; prophages paradoxically remain prevalent in bacterial genomes (9, 98). While the factors determining the loss or retention of prophages are not fully elucidated, recent findings propose that maintenance depends largely on host-conferred benefits under specific environmental conditions rather than on mutation alone. Bailey et al. (99) demonstrated that high induction rates, triggered by mitomycin C, accelerated prophage loss through strong directional selection. Conversely, under combined ampicillin and mitomycin C stress, prophage retention was prolonged when the prophage carried antibiotic resistance genes. Long-term experimental evolution, however, revealed a higher frequency of prophage loss coupled with the emergence of phage resistance and chromosomal ampicillin resistance mutations, suggesting parallel adaptation via selective sweeps to environmental stressors. Notably, stochastic simulations predicted that prophages carrying genes exclusively benefiting lysogens (e.g., efflux pumps) are retained longer than those providing community benefits (e.g., beta-lactamases), proposing that evolutionary trajectories of prophages depend on the type of conferred benefit (99). This evidence indicates that prophage retention is governed primarily by the net fitness balance between the metabolic cost of maintenance and the selective value of prophage-encoded functions under prevailing environmental conditions, with mutational drift playing a secondary role.

Beyond prophage loss, bacteria can alleviate the cost of prophage retention by regulating induction rates, prophage inactivation, mutation, and domestication (9, 100, 101). Since prophage carriage can be costly under certain conditions, inactivation is a frequent step in prophage evolution. MGEs, such as transposase genes and insertion sequences (IS), are significantly enriched in cryptic prophages, suggesting that they play an important role in inactivation (98, 100). Over time, these cryptic prophages undergo genomic degradation following complete or partial deletion, wherein beneficial genes are maintained by strong purifying selection (9). This model is supported by *in silico* genomic analyses in *Enterobacteriaceae* and other taxa, in which a bimodal length distribution was observed, consisting of larger prophages representing recent inactivation events, whereas shorter prophages reflect long-term genetic erosion or strong purifying selection (9, 100). Consistently, modeling studies showed that both negative and positive selection occur on cryptic prophages, resulting in the loss of re-infection and lytic genes (capsids, lysins, and terminases) and the retention of accessory genes that likely enhance host fitness (100). In some taxa, such as *Clavibacter*, most phage-like elements showed a trend toward short and inactive prophages, suggesting an advanced state of domestication (101).

Cyclical patterns of prophage loss and reacquisition are common among closely related lineages, indicating a continuous genetic flux possibly conditioned by local ecology (9, 99). Occasionally, some prophage-derived elements achieve full domestication and transition into essential host components, such as tailocins, gene transfer agents, or novel functional proteins (11, 102, 103), marking a definitive shift from parasitism to mutualism. Nonetheless, the precise molecular mechanisms and other critical factors driving prophage inactivation, degradation, and domestication remain to be fully characterized. Because prophage retention and loss are continuously determined by ecological pressures acting at the population and community levels, these dynamics must also be considered in the broader evolutionary consequences for microbial ecosystems.

## ECOLOGICAL AND EVOLUTIONARY CONSEQUENCES OF PROPHAGE INTERACTIONS

### Microbial population dynamics and evolution

One of the main ecological effects of temperate phages is the modulation of bacterial populations through alternated lysogenic-lytic cycles. Canonical models, such as Kill the Winner (KtW) and Piggyback the Winner (PtW), predict host-phage dynamics based on host cell density, explaining population oscillations as the result of lysis-lysogeny decisions (104). However, these models often overlook other factors like prophage-inducing agents (e.g., chemicals, environmental factors, and signal molecules), HGT, and other MGEs. Consequently, the Kill the Competitor model proposes that prophage induction can occur independently of cell density, wherein a subset of lysogens activates prophage production to trigger lysis of phage-sensitive competitors, allowing the remaining lysogenic population to thrive (105). This strategy is also exploited by bacteria that produce metabolites to induce prophages in competitors (106). Similarly, *in vitro* studies showed that prophages are induced by bile salts and hormones, highlighting a tripartite interplay mediated by eukaryotic hosts (107, 108). Meanwhile, bacteria can release outer membrane vesicles (OMVs) that function as decoys, capturing phages via receptor-mediated binding to avoid infection. Strikingly, OMVs also facilitate the transfer of phage receptors to phage-resistant strains (109). Furthermore, Silveira et al. (110) introduced the refugium hypothesis, in which coinfections and host metabolism determine the lysis-lysogenic decisions. At higher densities or low diversity, coinfections favor lysogeny due to inefficient metabolism for induction following PtW. At intermediate densities, encounter rates and metabolism favor lysis following KtW. Finally, at low densities and under starvation, lysogeny is extended, increasing coinfection frequency in line with the refugium hypothesis (110). In this context, phages are often considered drivers of microbiome composition either by directly killing or by indirectly releasing nutrients promoting the growth of other bacterial populations. Nonetheless, a recent trend proposes that phages may sometimes be followers rather than drivers of microbiome modulation, wherein the community context profoundly constrains the outcome of phage-host interactions (111, 112). Collectively, these models show that lysis-lysogeny decisions are context-dependent and influenced by the interplay of cellular density, environmental signals, metabolic state, and specific interactions (competence and evasion) within the bacterial community and eukaryotic hosts.

Cyclic dynamics in phage-host populations can also emerge from the lysis and regrowth of coevolved host and phage populations. In this scenario, hosts develop resistance to phage infection, and phages consequently acquire counter-mutations driving arms race or Red Queen dynamics (112). The arms race involves a directional and continuous increase in resistance and infectivity that replaces ancestral populations (113). In contrast, the Red Queen model describes a long-term non-directional coevolution where defense and infection variants oscillate within the population due to fluctuating selection, thereby increasing genetic diversity (114, 115). Although phage resistance is typically attributed to phage-driven accelerated mutation rates (50), the King of the Mountain hypothesis suggests that dominant resistant populations can instead arise through high levels of recombination via HGT and CRISPR spacer acquisition rather than only phase variation and receptor modification (112, 116). Since all these oscillations are accompanied by genetic changes on similar timescales, they exert reciprocal effects on ecological processes, leading to eco-evolutionary dynamics (117). In this context, the rapid coevolution may mirror KtW patterns but is mediated by phage resistance rather than cell density alone. Ultimately, the coevolutionary outcome depends on selective pressures and fitness costs associated with resistance. Directional selection during the arms race increases both resistance and infectivity, often imposing higher resistance costs in fluctuating environments. In contrast, fluctuating selection maintains diversity through negative frequency-dependent selection (112). These models are not mutually exclusive; indeed, evidence suggests that coevolution often initiates with arms race dynamics and subsequently shifts toward fluctuating selection as resistance costs accumulate and ecological inputs (e.g., spatial heterogeneity, changes in cell density, and environmental factors) alter the selective landscape (118). Consequently, phage-host interactions can follow divergent scenarios: boosting the acquisition of niche-specific traits that foster speciation or the emergence of ecotypes through asymmetric coevolution or leading to the extinction of either the phage or host populations (112). These coevolutionary processes demonstrate that phage-host interactions fail to adhere to a single trajectory; instead, the result is contingent upon the interplay of selection forces, the fitness costs associated with resistance, and the ecological complexity of the environment.

Most ecological and evolutionary models focus primarily on temperate phages and, to a lesser extent, lytic phages. However, the role of cryptic prophages has been frequently overlooked, despite their contribution of genetic features that confer competitive advantages. Temperate phages can also recombine with these cryptic elements, promoting gene shuffling and mosaicism, which accelerates phage evolution (8, 45). Moreover, cryptic prophages might be reactivated through recombination with superinfecting or co-resident active phages. Regarding lytic phages, evolutionary studies indicate that specific conditions, such as small habitat size, can increase phage extinction rates (119). Furthermore, most ecological and coevolutionary models are limited to single phage-host pairs. In nature, however, hosts are predated upon by multiple phages, and phages often exhibit broad host ranges. This complexity opens the need for network analysis to accurately weigh the impact of local adaptation (113). Other critical factors include immigrant phages, community structure, encounter rates, multisensitization following an initial viral infection, and unequal environmental pressures (120, 121). Given the sensitivity of phage-host dynamics, more integrative approaches are required to understand coevolution within complex microbial communities. While prophages modulate the microbiome through lysis-lysogeny decisions and resistance, lysogens may enhance their fitness by acquiring adaptive traits, as discussed in the next section.

### Niche diversification and adaptation

Beyond their role in population dynamics, prophages profoundly impact bacterial phenotypes through mechanisms that confer competitive advantages to lysogenic populations. As previously discussed, prophages serve as reservoirs of diverse genetic elements that influence host phenotypes at multiple levels, from the expression of novel genes to transcriptional modulation via sRNAs, transcriptional factors, and promoter activation (Tables 2 and 3). The impact of these mechanisms varies according to niche-specific selective pressures, fitness costs, and the degree of host-phage coevolution. In some instances, these tight interactions facilitate the coevolution of multiple partners, as seen in tripartite phage-microbe-eukaryote interactions.

At the ecological level, a primary advantage is superinfection immunity, which protects lysogens from related viral infections (7). Additionally, prophages enhance host fitness to outcompete others for space and resources or increase survival under harsh conditions, thereby favoring lysogenic populations and promoting niche adaptation and diversification (7, 120). For example, prophage genes promoting host survival against heavy metals, antibiotics, or low nutrient conditions facilitate colonization of new environments (51, 69, 71, 72). Meanwhile, morons can drive the emergence of highly infectious pathogenic strains (52). Prophages also mediate other symbiotic interactions. Examples include tail-like proteins in *Burkholderia gladioli* that facilitate endophytic colonization (122); ankyrin protein expression by microbial hosts colonizing sea sponges (67), or the precise restoration of host genes via active lysogeny to aid colonization, immune evasion, mutability, and sporulation (79, 83, 123). In certain scenarios, prophages can provide population-level benefits where non-lysogens act as cheaters benefiting from the public goods produced by lysogens (e.g., β-lactamases, biofilm exopolysaccharides) (99, 124). This phenomenon is further reflected by the complex cooperative interactions observed within polymicrobial colonies and biofilms (124). This indicates that prophages serve as conditionally activated genetic toolkits that enhance the ecological diversity of their hosts by contributing niche-specific adaptive traits, facilitating both niche specialization and the emergence of novel bacterial ecotypes.

The impact of lysogens extends to the regulation of global processes, such as the biogeochemical cycles, through the transformation of carbon and phosphorus, the recycling of organic matter, and the production of novel compounds via AMGs (72, 125). Furthermore, lysogeny facilitates the diversification of phage and bacterial populations, enriching genetic reservoirs of diversity (45, 49, 124). A definitive example of this evolutionary impact is the co-option and domestication of prophage genes, such as *SpmX*, which gave rise to an entirely new bacterial clade (11). These adaptive advantages are modulated by bacterial immune systems, particularly CRISPR-Cas, which can influence prophage acquisition, persistence, and activity.

### CRISPR-Cas interactions

The primary role of bacterial defense systems is to antagonize viral infections; however, they may also impact the host genome by restricting HGT. Comparative genomic studies revealed that bacteria harboring CRISPR-Cas subtypes and other antiphage systems often possess reduced genomes characterized by a lower gene acquisition rate, smaller pangenomes, and fewer phage-related elements (39, 42). In *Staphylococcus epidermidis* and *P. aeruginosa*, CRISPR-Cas systems limit the acquisition of plasmids and phage DNA by targeting and degrading foreign genetic material (114, 126). Although these barriers prevent the entry of potentially deleterious elements, they also restrict the acquisition of beneficial genes, thereby potentially reducing bacterial fitness. For instance, in *B. thuringiensis*, the introduction of a heterologous active CRISPR-Cas system from *B. cereus* resulted in impaired sporulation, reduced pathogenicity, and diminished tolerance to salt and pH stress, suggesting that constitutive CRISPR-Cas activity can restrict environmental adaptability and distribution (127). This establishes that while CRISPR-Cas systems protect against potential genetic invasion, they simultaneously restrict the acquisition of elements derived from beneficial prophages, which could limit genomic plasticity.

Conversely, emerging evidence suggests that CRISPR-Cas systems act as a double-edged sword, potentially accelerating phage evolution and host genetic diversity by facilitating mutation, HGT, recombination, and silencing. CRISPR-Cas9 pressure has been shown to increase the mutation rate of phage T4 by approximately six orders of magnitude, driving rapid viral diversification (115). Strikingly, CRISPR-Cas may promote HGT in specific contexts. In *P. atrosepticum*, CRISPR-Cas impeded the transduction of plasmids and pathogenicity islands while enhancing the generalized transduction of CRISPR-Cas loci among phage-sensitive subpopulations (128). Additionally, phages can recombine with CRISPR spacers, facilitating their dissemination via specialized transduction in *S. aureus* and *S. pyogenes* (48, 129). Varble et al. (129) demonstrated that staphylococcal phages (e.g., phage 85, ΦNM1, ΦNM4, and Φ12) recombine with chromosome or plasmid-borne CRISPR arrays, leading to the lateral transfer of CRISPR-adjacent genes. Similar findings were observed in phage ΦAP1.1, which expresses an anti-CRISPR (Acr) protein to inhibit Cas9, allowing its integration into the CRISPR locus of *S. pyogenes*. Upon mitomycin C induction, imprecise excision of the ΦAP1.1 prophage triggered the packaging of adjacent CRISPR spacers into virions spreading immunity to new hosts. Interestingly, ΦAP1.1 integration selectively neutralized host immunity by leaving active spacers that target phage competitors while retaining the ability to incorporate new spacers, broadening the repertoire of viral targets (48). Beyond defense, CRISPR-Cas can regulate cryptic prophages through a mechanism analogous to RNA interference (RNAi), where crRNAs bind to prophage mRNAs to silence deleterious genes. In *E. coli* K-12, CRISPR-Cas inactivation or deletion led to growth inhibition and cell death associated with the increased transcription of cryptic prophages (e.g., DLP-12, Rac, Qin, and CP4-57), suggesting that CRISPR-Cas is essential for stable coexistence with these elements (130).

The interplay between phages and CRISPR-Cas has profound implications for genome plasticity. By increasing mutagenesis, HGT, recombination, and prophage silencing, CRISPR-Cas may expedite coevolutionary arms race dynamics (131). These findings suggest that CRISPR-Cas systems did not only evolve solely to avoid phage predation but also to provide fitness benefits to both the phage and the host. Ultimately, the evolutionary outcome of CRISPR-Cas and phage interactions depends on the fine balance between protection from infection and the adaptive cost of losing prophage-derived benefits (127, 132).

The coevolutionary dynamics between prophages and CRISPR-Cas systems represent a node within a broader network of phage-bacteria interactions. These viral elements are also involved in a competitive and cooperative relationship with plasmids, PICIs, and insertion sequences, which likewise influence prophage evolution.

### Interaction and coevolution with other MGEs

Phage-host coevolutionary dynamics are shaped by competitive and cooperative interactions with other MGEs, such as prophages, PICIs, plasmids, and IS. In polylysogens, competition between co-resident prophages is driven by factors that maximize host resource monopolization and increase viral production, including shortened latency periods and competitive inhibition (23, 133). For instance, LambdaSo inhibits MuSo2 replication during planktonic growth under mitomycin C induction, although both prophages exhibit similar levels of production during biofilm formation (23, 134). In *Salmonella* intra-macrophage persisters, the RemAIN element within prophage Gifsy-1 specifically blocks the induction of co-resident prophages (133). Conversely, prophages can interact synergistically. In *V. cholerae*, the RS1 element forms hybrid virions with CTXφ prophages via chromosomal replication, whereas solitary CTXφ rarely produces virions (46). Phage satellites, such as PICIs, parasitize helper phages by hijacking capsids or tails for genome packaging, thereby interfering with helper reproduction (135, 136). PICIs also influence phage-host dynamics by reducing lysogenization, increasing cell survival, and promoting population genetic heterogeneity in transduced cells (137). Moreover, some PICIs and prophages encode diverse antiphage defense systems to prevent superinfection, limiting infection of other phages (40, 43, 138). These prophage interactions reveal that co-resident MGEs are organized into competitive and cooperative networks that shape phage production, host survival, and the evolutionary trajectories of all participating elements.

Phages establish more diversified interactions with plasmids. Phages promote the dispersal of small plasmids via generalized transduction (35), exploit conjugative plasmid proteins as receptors (139, 140), limit plasmid transfer (140, 141), stabilize plasmid maintenance (19), and protect co-resident plasmids from superinfection (141). Conversely, plasmids can restrict phage host range and infectivity by reducing receptor expression (142), providing defense genes such as toxin-antitoxin systems (143), or suppressing spontaneous prophage induction (19). Additionally, IS have been associated with early prophage inactivation (100). In *Helicobacter pylori*, IS act as recombination hotspots, driving genomic rearrangements and mosaicism that enhance both prophage and host diversity (144).

These inter-MGE interactions establish an intricate network that profoundly influences phage-host coevolutionary trajectories. Antiphage activities of satellites, co-resident prophages, and plasmids shape inter-viral competition and the selective pressures imposed by phage predation. Notably, defense systems carried by PICIs may elucidate the origin and evolution of bacterial defense islands (43). Furthermore, antagonistic plasmid interactions may drive the parallel evolution of plasmid-phage pairs toward plasmid-dependency (142). While conjugative plasmids transferred across distantly related taxa can broaden phage host range (145), phages may also mobilize non-transmissible plasmids via generalized transduction, contributing to interspecies and intergeneric mobilization (35). Ultimately, these interactions enhance genomic plasticity and the dissemination of fitness-conferring genes, driving genomic heterogeneity within host populations and the emergence of novel genetic combinations.

## CONCLUDING REMARKS

**Fig 2:**
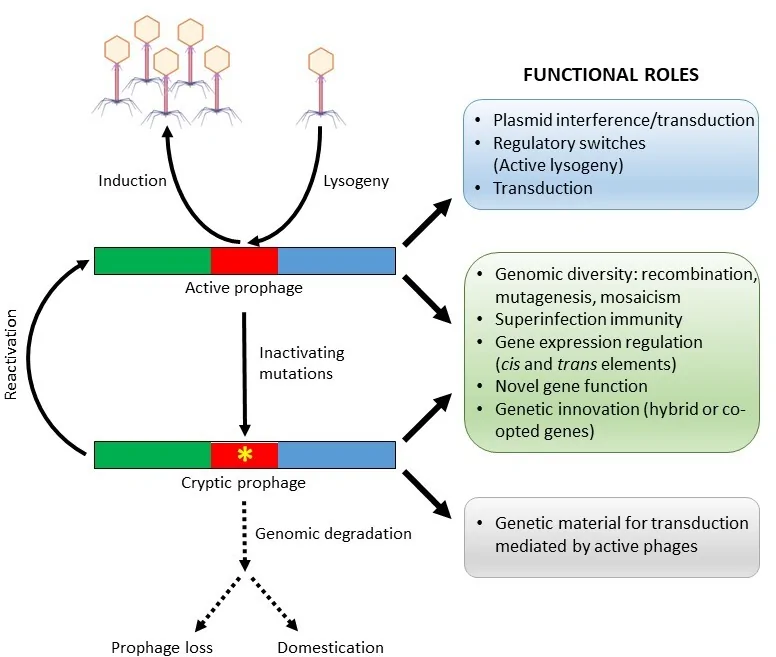
Summary of functions mediated by active and cryptic prophages. Active and cryptic prophages possess overlapping functional roles that mutually influence host phenotypes. Active prophages can be induced by various stimuli through alternating lysogeny-lytic cycles, triggering host population fluctuations. During lysogeny, active prophages provide functional and regulatory diversity through their own genes and genetic material transduced from other hosts and MGEs, thereby increasing genomic plasticity and fitness. Notably, active prophages can also drive plasmid evolution through interference and transduction. In contrast, although cryptic prophages cannot be induced, they play relevant roles in gene function and regulation similarly to their active counterparts. Cryptic prophages often undergo genomic degradation, which may result in either the complete loss or the domestication of beneficial genes. Furthermore, active prophages can become cryptic through specific mutations that impair excision function. Conversely, cryptic prophages may occasionally reactivate through recombination or compensatory mutations that restore the inducible module. Figures were created with BioRender/Canvas.

Prophages are dynamic components that influence the host genome structure, functionality, and interactions with other microorganisms (Fig. 2). However, their full impact on bacterial ecology and evolution remains far from being understood. Most studies have focused on phage-host pair interactions, but current studies demonstrate that coevolutionary dynamics depend heavily on intrinsic elements (e.g., prophages, plasmids, and other MGEs) and extrinsic factors (e.g., microbial community structure and environmental conditions). These factors are critical determinants that alter ecological functions within microbial communities, with profound effects on evolutionary timescales. Furthermore, a significant gap remains between prophage prediction and gene function due to limitations in identifying prophage boundaries, the mechanisms driving prophage domestication or loss, and the roles of uncharacterized “dark matter” proteins. Dissecting the fundamental mechanisms of phage-host interplay during adaptation and evolution will expand our understanding of how microbial communities evolved and diversified. This knowledge also holds practical applications. For instance, understanding antagonistic interactions between prophages is essential when developing therapeutics against antibiotic-resistant pathogens using temperate phages. Moreover, the presence of specific plasmids can critically regulate phage infectivity and host range, directly impacting the efficacy of phage therapy (142). Thereby, expanding our knowledge of viral interactions, ecology, and evolution will be pivotal for exploiting their potential in biotechnology and medicine.
